# Weighing trees with lasers: advances, challenges and opportunities

**DOI:** 10.1098/rsfs.2017.0048

**Published:** 2018-02-16

**Authors:** M. I. Disney, M. Boni Vicari, A. Burt, K. Calders, S. L. Lewis, P. Raumonen, P. Wilkes

**Affiliations:** 1 UCL Department of Geography, Gower Street, London WC1E 6BT, UK; 2 NERC National Centre for Earth Observation (NCEO), UK; 3 Earth Observation, Climate and Optical Group, National Physical Laboratory, Teddington TW11 0LW, UK; 4 School of Geography, University of Leeds, Leeds LS2 9JT, UK; 5 Tampere University of Technology, Laboratory of Mathematics, Korkeakoulunkatu 10, 33720 Tampere, Finland

**Keywords:** above-ground biomass, terrestrial laser scanning, lidar, canopy, structure, buttress

## Abstract

Terrestrial laser scanning (TLS) is providing exciting new ways to quantify tree and forest structure, particularly above-ground biomass (AGB). We show how TLS can address some of the key uncertainties and limitations of current approaches to estimating AGB based on empirical allometric scaling equations (ASEs) that underpin all large-scale estimates of AGB. TLS provides extremely detailed non-destructive measurements of tree form independent of tree size and shape. We show examples of three-dimensional (3D) TLS measurements from various tropical and temperate forests and describe how the resulting TLS point clouds can be used to produce quantitative 3D models of branch and trunk size, shape and distribution. These models can drastically improve estimates of AGB, provide new, improved large-scale ASEs, and deliver insights into a range of fundamental tree properties related to structure. Large quantities of detailed measurements of individual 3D tree structure also have the potential to open new and exciting avenues of research in areas where difficulties of measurement have until now prevented statistical approaches to detecting and understanding underlying patterns of scaling, form and function. We discuss these opportunities and some of the challenges that remain to be overcome to enable wider adoption of TLS methods.

## 1. Introduction

In the century since the publication of D'Arcy Wentworth Thompson's classic text *On Growth and Form* [1] measurements of organism size, mass and form have become central to quantitative ecology. In the case of trees, the size of an individual, its above- and below-ground biomass, and the relationships between them, or allometry, are key properties of interest. The biomass represents the accumulated productivity of the tree in terms of stored carbon (C) and, as a result, quantifying above-ground biomass (AGB) of trees on large scales is vital in order to estimate C stocks and fluxes resulting from deforestation, degradation and regeneration [2].

Estimating the total mass of C stored in a tree requires measuring both the above- and below-ground (root) biomasses, via harvest and weighing. Measuring either of these two masses is difficult, time-consuming and expensive in practice, as well as being, by definition, destructive. AGB is the more widely measured^1^ property [3,4], in large part, due to the relative ‘ease’ of measurement compared with the below-ground component. Below-ground biomass is extremely difficult to measure and as a result tends to be poorly quantified, inferred as it is from proxy observations and models, calibrated and validated using very limited samples of real biomass [5]. Direct measurement of AGB also requires destructive harvesting [6], and the difficulty of achieving this increases in remote or inaccessible regions, particularly large parts of the tropics. Destructive harvesting also precludes repeat measurements to capture dynamics, is often undesirable in the case of endangered, old growth or arboretum specimen trees, or may be prohibited outright in protected areas. Consequently, estimates of AGB at the tree and plot scale rely, by necessity, on indirect methods, namely empirical size-to-mass allometric scaling equations (ASEs) [6–11]. ASEs are based on compilations of destructive harvest measurements made of relatively few, mostly smaller trees. Trees are very often selected for harvest by loggers and so are rarely selected systematically with the resulting ASE derivation in mind [6,12,13]. This results in ASEs which have potentially large and unknown bias [14–16].

Estimating AGB accurately is critical for several reasons [17,18]. First, it forms the basis of estimates of the largest terrestrial C stocks and fluxes [2,9,19,20]. Forests hold 70–90% of terrestrial above- and below-ground biomass [21], but estimates of the amount and distribution of this biomass are based on a small number of poorly distributed samples, poorly distributed spatially and with potentially large biases. These uncertainties arise due to, *inter alia*, undersampling of the species-rich tropics in comparison with temperate and boreal region [22,23]; lack of harvest measurements of large trees [6,24]; and the form of the ASEs used to predict AGB [13,22,25]. The terrestrial carbon sink, the residual of the net gains and losses between the biosphere and atmosphere, has increased over the last two decades [20], but the measurement uncertainties mean that the magnitude, location and causes of this residual terrestrial sink are still not well quantified [21].

Second, large differences arise between estimates of both AGB stocks and consequent deforestation fluxes, particularly in magnitude, but also location [17,23,26–29]. Some of these discrepancies are attributable to definitional and methodological differences [19], but much uncertainty remains, particularly over spatial distribution of the residual terrestrial sink [17].

Third, AGB is a key component of terrestrial ecosystem function, as part of the more general energy, nutrient and hydrological cycles. Estimates of AGB are required to test land surface process models (LSMs) which predict (or are calibrated against observed) AGB, as part of understanding and forecasting ecosystem processes [30,31].

Finally, accurate (or at least precise) estimates of AGB with quantified uncertainty underpin international efforts to mitigate impacts of climate change [32]. Forests are earmarked to provide one-quarter of planned greenhouse gas emission reductions under the United Nations Paris Agreement on Climate Change [33,34]. Current discrepancies in terms of tropical forest biomass alone are as much as 45.2 Gt C, valued at US$1 trillion [35,36].

## 2. Measuring above-ground biomass

Since the early 1980s, significant advances have been made in estimating forest AGB, particularly from remote sensing. These have been used to augment standard forest inventory approaches, primarily aimed at estimating merchantable timber quality. Forestry estimates of AGB typically involve manual plot-level measurements of diameter-at-breast height (DBH), which are then converted to AGB (or timber mass) via allometry and then upscaled via forest area [37,38]. These methods are relatively easy to make, repeatable and transferrable; uncertainty arises due to the allometry and upscaling process. Remote sensing has allowed wide-scale, accurate estimation of forest cover change [39], which can be converted to C gains/losses via inventory data on C density [33,40]. The advent of airborne and spaceborne lidar has allowed allometric estimates of AGB derived from canopy height and density metrics [19,26,27,41,42].

However, all these methods are indirect: they rely on extrapolating very limited harvest AGB data via some combination of forest cover, C density and height/diameter allometry. The often very different assumptions, on which these indirect estimates are based, lead to the following problems: (i) they are very hard to validate in any meaningful way [16]; (ii) it is difficult to compare them, or to reconcile differences when they are compared [28,29]; (iii) uncertainties are poorly quantified or even unknown [16]. Terrestrial laser scanning (TLS) has the capability to address these problems, by providing tree- and plot-level AGB estimates which are independent of allometry, unbiased in terms of tree size distributions and with well-quantified uncertainty. TLS estimates collected widely and reliably can reduce current uncertainties in terrestrial C stocks, enable improved calibration and validation of AGB products, particularly from remote sensing, and form the basis of improved allometric models. Here, we describe key developments in the use of TLS to estimate AGB, present analysis of uncertainties that should be addressed, and highlight challenges that remain.

## 3. Terrestrial laser scanning-derived estimates of above-ground biomass

Data used for analysis are deposited in the dryad database (http://dx.doi.org/10.5061/dryad.02dq2) [43]. The TLS data underlying the three-dimensional (3D) models presented here were all collected using a Riegl VZ-400 TLS instrument, following protocols developed using the experience gained during various field campaigns in the tropics and elsewhere. In general, 1 ha plots were scanned on 10 or 20 m grids, with individual scans co-registered via static reflectance targets into a single large point cloud for each 1 ha plot. These methods are described in detail in [44–50]. The Riegl instrument is towards the upper end of the cost for TLS (in the £75–150 K range depending on model and accessories), with a range of approximately 700 m and a pointing accuracy of millimetres at that range, as well as waveform capabilities. An increasingly wide range of TLS instruments are now available, costing from approximately £10 K upwards, with increased cost generally corresponding to increased range and potentially also accuracy (but also reduced size, robustness, increased functionality etc.). The advantages and disadvantages of some of these various systems are discussed in [47] and [48].

### 3.1. Information content of terrestrial laser scanning data

**Figure 1.**
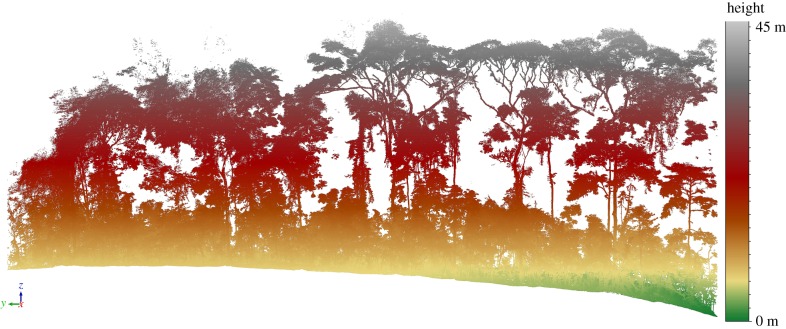
70 × 5 m transect of TLS data collected in Ankasa, Ghana, March 2016, with points coloured by height. The data are from a 70 × 100 m plot which was scanned with two Riegl VZ-400 TLS instruments, using a 10 m grid spacing between scan locations as described in [48]. The plot contained 270 trees with DBH greater than 10 cm, with TLS-estimated AGB of 234 tons (or approx. 334 t ha^−1^).

**Figure 2.**
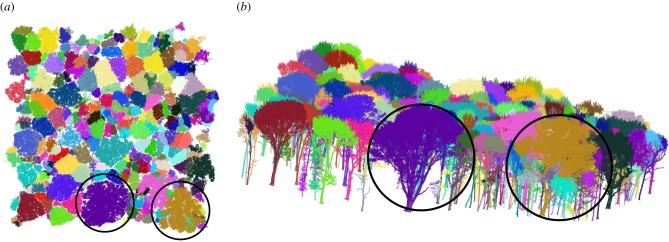
Terrestrial laser scanner data from deciduous woodland, Wytham Woods, UK, showing separate tree point clouds, and example trees used in the analysis below. (*a*) Plan view of 1 ha TLS point cloud. The extracted point cloud of each tree is coloured separately. The sycamore (*Acer pseudoplatanus*, left) and ash (*Fraxinus excelsior*, right) trees analysed below are circled. (*b*) Oblique view, with the sycamore and ash trees again circled.

**Figure 3.**
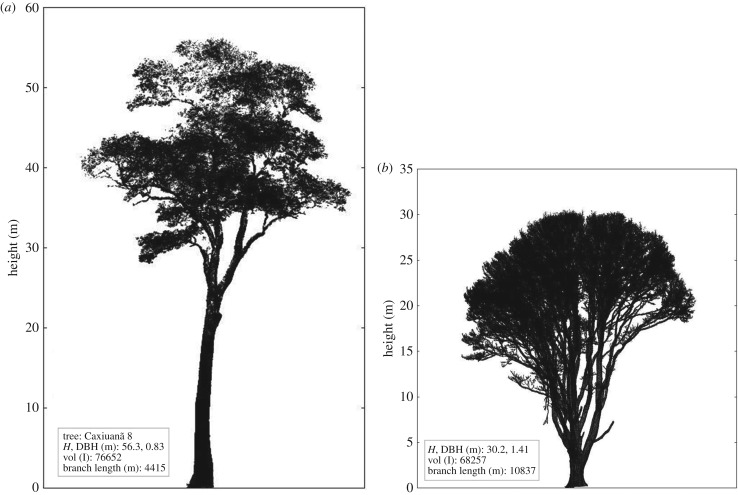
(*a*) Caxiuanã hardwood scanned leaf-on, 2013. (*b*) Wytham sycamore, scanned leaf-off (downsampled to 0.026 m point spacing), 2015. In each case the height, DBH, volume and total branch length of the tree are given; note the different vertical scales in each case.

Figures 1–3 show examples of the rich information content of TLS data. Figure 1 shows a 70 × 5 m transect through a larger forest plot scanned in Ghana, West Africa. Figure 2 shows 1 ha of TLS data collected in Wytham Woods, near Oxford, UK (http://www.wytham.ox.ac.uk/), an extensively studied area of deciduous woodland. These data are part of a larger 6 ha region scanned in leaf-on and leaf-off conditions during 2015 and 2016, as far as we know the largest single area scanned with TLS in this detail [44,45]. Figure 3 shows point clouds of two individual trees extracted from larger, plot-scale point clouds collected in two contrasting forest environments: tropical rainforest in Brazil and Wytham Woods. It is notable that the sycamore tree (*Acer pseudoplatanus*) in the latter case has an extraordinary 10.8 km of branch material! This is more than double that of the tropical tree, which is 25 m taller. The two trees have broadly comparable volumes despite their different height and shape, but their resulting AGB will depend on wood density, *ρ* (discussed below). These figures highlight both the extraordinary diversity of tree form and the ability of the TLS data to capture and quantify this diversity, not just for estimating AGB but also to address fundamental questions about the relationship between tree form and function [51,52].

### 3.2. Three-dimensional tree structure and volume from terrestrial laser scanning

We outline some of the key uncertainties in TLS-derived estimates of AGB below. The approach we focus on here is that of quantitative structural models (QSMs) [53–58]. Various other approaches to estimating the volume of tree components also exist, notably focusing on the main trunk, or volume-based fitting to the trunk and crown as a single geometric shape [59,60]. These methods have generally been developed for forestry applications, for lidar instruments with range less than a few tens of metres, or for single-scan acquisition, i.e. where co-registration of multiple TLS acquisitions is either not feasible or not desirable [60]. Forestry-based TLS approaches are reviewed by Thies *et al*. [59] and Niklas [61]. However, QSMs are currently the most accurate way to estimate tree volume, AGB and structure [25,59].

**Figure 4.**
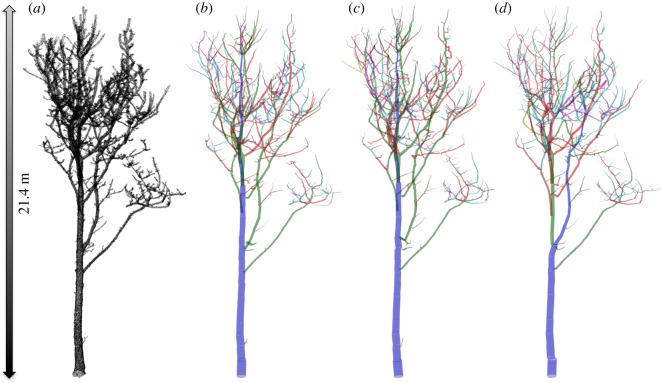
TLS point cloud of a single tree, scanned leaf-off (*a*), and three instances of fitting a QSM to the cloud (*b*–*d*), using the approach of Raumonen *et al*. [53]. Each QSM uses the same parameters, but a different starting seed value, resulting in slightly different reconstructions in each case. The colours represent the branching order within the model.

Prior to applying any volume-extraction algorithm, multiple individual scans, either around a single tree or through a larger area, need to be accurately co-registered and merged into a single point cloud [46]. Following this, the QSM approach relies on fitting geometric primitives such as cylinders, or even a tessellated mesh surface, to the lidar point cloud of a single tree, to obtain a closed volume of 3D tree structure. This process encompasses various possible stages and assumptions [49,50,53–58]. Figure 4 shows the progression of a tree from a point cloud, through three iterations of QSM fitting using the approach of Raumonen *et al*. [53] modified by Burt [50].

The resulting QSMs provide topologically connected, enclosed volumes [49,50,53–58] comprising the volume of all individual trunk and branch components. The size, position, orientation and ordering of branches (parent–child distributions) is key information for many ecological applications, particularly testing predictions of metabolic scaling theory [51,61–64]. Here, we focus on some of the uncertainties and challenges in using TLS-derived QSMs for estimating AGB and suggest possible developments and strategies to address some of these uncertainties.

### 3.3. Quantitative structural model volume uncertainty

Uncertainty in QSM volume arises broadly from limitations of the TLS measurements and inherent uncertainty of the QSM reconstruction process. Branches of similar diameter (or smaller) to the TLS footprint at a given distance are not generally resolved in sufficient detail to be reconstructed accurately. This occurs more frequently higher in the canopy, where occlusion is exacerbated by higher cover and by the larger distance from the instrument. TLS pulses reaching the uppermost part of the canopy do so with larger footprint, depending on the instrument beam divergence. For the current commercial TLS instruments (e.g. Riegl http://www.riegl.com/products/terrestrial-scanning/, Leica http://leica-geosystems.com/en-gb/products/laser-scanners, Faro http://www.faro.com/products/3d-surveying/laser-scanner-faro-focus-3d/overview) the footprint is of the order of 2–5 cm at 100 m and hence branches of less than approximately 5 cm diameter will be poorly resolved at this distance. This results in greater uncertainty in estimated volume, albeit only for a small fraction of the total ([49]; note that 80% of AGB in their study is contained in the lower 60% of plot height). Other uncertainties inherent in TLS data include: wind disturbance, a random error which is minimized by scanning during calm conditions wherever possible; and co-registration accuracy. The latter is determined by the instrument properties and by the ability with which specific targets or features can be identified in multiple scans. With care, co-registration accuracy can be close to the range accuracy (4 mm) of the instrument over 1 ha [46].

The inherent uncertainty of QSM reconstruction can be further sub-divided into a stochastic component, arising from the need for non-deterministic numerical procedures for fitting shapes, lines etc. to regions of the point cloud, and a systematic component arising from the assumptions underlying a particular QSM approach. For example, the use of cylinders as geometric primitives may lead to volume overestimation due to localized branch tapering [59]. These errors tend to increase in a relative sense with decreasing branch size, but the resulting impact on absolute volume (and AGB) decreases correspondingly with branch size.

There are also choices of parameters to be made during reconstruction, particularly the size of region that geometric objects are fitted to—*d*_min_, the diameter of the patch used to fit to a point cloud region in the TreeQSM^2^ [53,55]. In addition, the point cloud is partitioned into regions in random order, so QSM volume varies even for a fixed parameter set, and a given QSM should therefore be viewed as a sample from a distribution of possible volumes (as illustrated in figure 4). In practice, QSM generation is generally carried out multiple times for a given tree point cloud to provide a final volume estimate with an associated estimate of uncertainty [25,50].

### 3.4. Irregularity of tree form

Uncertainty in allometric estimates of AGB arises (in part) from the fact that many trees have irregular, hollow or damaged trunks, or feature buttressed trunks, particularly in large tropical trees [65–67]. Buttresses may be accounted for implicitly in ASEs through inclusion in harvest data [67] or explicitly by considering trunk form, but the resulting ASEs provide only unbiased biomass predictions for forests with a similar proportion of buttressed trees to those sampled. Corrections have been proposed to account for the impacts of buttressed trees in ASEs [65,67,68], but these rely, in turn, on destructive harvesting of an even more uncertain population (irregular trees).

Trees with irregular trunk shape and form also affect the accuracy of AGB estimation via QSM reconstruction. Buttressed trunks tend to be far from cylindrical, at least close to the ground, potentially with significant biomass in this lower portion. Examples of this can be seen in figures 1 and 3. QSM fitting assumes (in general) that cylindrical sections can be fitted around the TLS point clouds. However, QSM reconstruction is also possible using mesh grids to fit an enclosing surface to an arbitrarily defined lower part of the trunk point cloud. Below, we compare the volume of some complex tree trunk shapes estimated using a closed triangulation surface model with volumes estimated from TreeQSM cylinder fitting.

**Figure 5.**
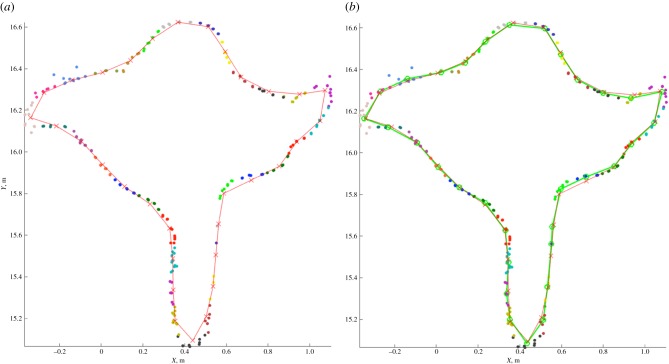
Generation of a new cross-section curve based on the previous curve. (*a*) Partition of the section based on previous vertices (red). (*b*) New curve (green) formed as averages of partition points.

The triangulation approach developed from Raumonen *et al*. [53] fits curves made of short line segments to thin horizontal sections of the TLS data. Figure 5 shows TLS points from a trunk with an exaggerated buttress, along with an example cross-section fitted with line segments. These curves model the cross-sections of the trunk, and the vertices of the curves in successive layers are then connected systematically to form a continuous triangulated surface. Finally, the top and bottom planes are triangulated to close the model. Initially, a horizontal cut plane is selected manually from the TLS data and the points below the plane are used for the triangulation. The level of the cut plane is arbitrary, but could be selected (for example) where the trunk cross-section is approximately circular, where the stem bifurcates into many large branches, or above a buttressed root system. In the large Wytham Woods sycamore, in figure 3, for example, the stem bifurcates near the ground, so the triangulated part is small compared with the whole tree/stem; this part may be much larger in other species and/or environments.

**Figure 6.**
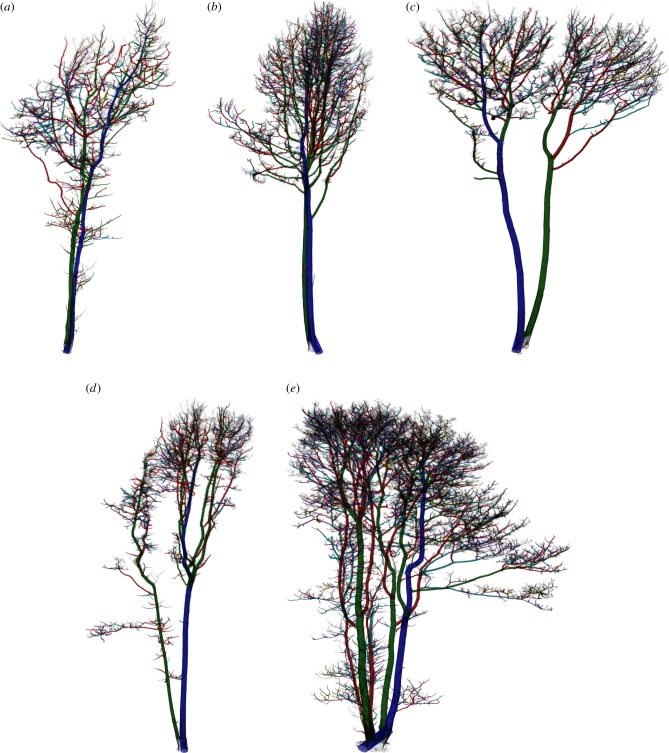
Trees used in the analysis of volume uncertainty in cylinder fitting. All trees are sycamore (*Acer pseudoplatanus*) from Wytham Wood. The height, DBH, branch length, volume and AGB of the resulting QSMs derived from these are given in each case (using *ρ* taken from http://www.wood-database.com/sycamore-maple/) are as follows. (*a*) 1009: *H* 21.2 m, DBH 0.22 m, *L*_branch_ 680 m, *V*_tot_ 1550 l, AGB 0.95 t. (*b*) 213: *H* 24.9 m, DBH 0.49 m, *L*_branch_ 1350 m, *V*_tot_ 4600 l, AGB 2.83 t. (*c*) 138: *H* 23.5 m, D 0.47 m, *L*_branch_ 1000 m, *V*_tot_ 4200 l, AGB 2.57 t. (*d*) 1570: *H* 25.8 m, D 0.47 m, *L*_branch_ 760 m, *V*_tot_ 2890 l, AGB 1.77 t. (*e*) 255: *H* 26.3 m, D 0.53 m, *L*_branch_ 2600 m, *V*_tot_ 8370 l, AGB 5.15 t.

**Figure 7.**
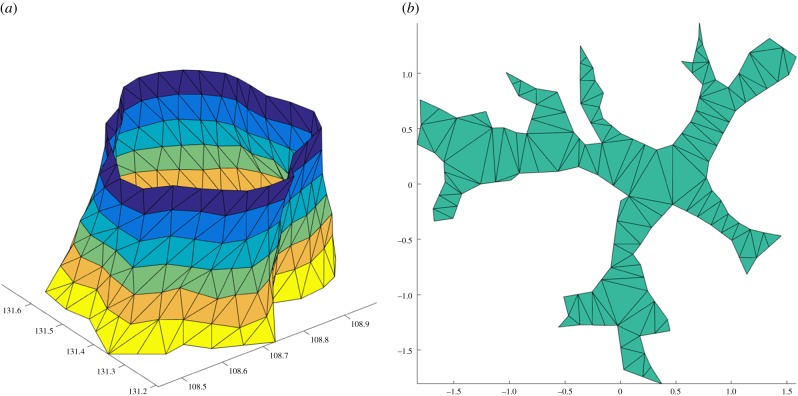
Generation of triangulation from the cross-section curves. (*a*) Side triangles reconstructed from successive curves. (*b*) Bottom plane.

**Figure 8.**
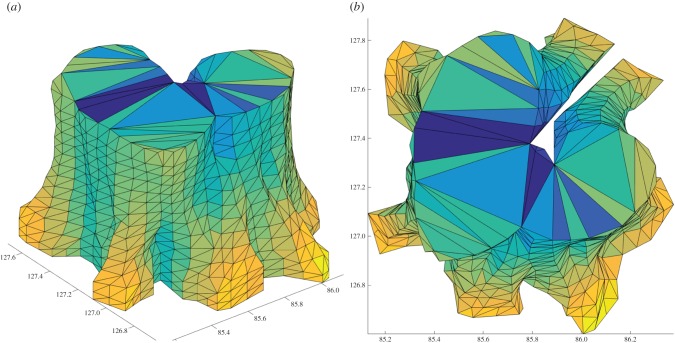
Final triangulation model from side (*a*) and top (*b*).

Once the first cross-section curve is defined, further cross-section curves are defined based on the previous curve, assuming successive cross-sections differ only slightly each time (figure 6*a*). Thus, the previous curve is translated vertically to the same level as the next cross-section and its vertices partition the points of the sections into the same number of disc-like neighbourhoods, whose means define the new vertices (figure 6*b*). This process of defining new cross-section curves stops when there are no more points in the point cloud, or when most of the vertices in the curve are translated vertices from the above curve, or there is a self-crossing curve. Delaunay triangulation is used on the top and bottom planes to close the model. Figure 6 shows the example trees extracted from the Wytham Woods data in figure 2; figures 7 and 8 show the final triangulation model.

Volume is computed from the final triangulation model using the divergence theorem, which requires the outward surface normal and area of each triangle. We compared the volumes of the triangulation model and QSM-derived stem cylinder for five trees from the Wytham Woods data in figure 6. The results are shown in table 1. The resulting triangulated mesh volumes are compared with their QSM cylinder-fitted counterparts, and the difference is shown relative to the full QSM tree volume in each case. For the lower trunk, differences could be as high as 45% (tree 1009), but because of under- and over-estimation between trees, the total difference over five trees was only 7.5%. Trunk volume differences compared with total QSM tree volume ranged from 0.2% to 1.6%.

**Table 1.** Volume in litres of lower trunk/root sections calculated from triangulation (*V*_tri_) and QSM cylinder fitting (*V*_cyl_) with 2*σ* in each case; the absolute difference between the two estimates; and the relative difference as a fraction of QSM volume, *V*_tot_.

tree	*V*_tri_ (l)	2*σ* (l)	*V*_cyl_ (l)	2*σ* (l)	*V*_cyl,tot_ (l)	2*σ* (l)	*V*_tri_−*V*_cyl_ (l)	*V*_tri_−*V*_cyl_/*V*_cyl,tot_ (%)
1009	63.5	2.5	34.8	5.8	1784	89	28.7	1.6
213	262.7	16.4	274.4	32.7	4606	153	−11.7	−0.3
138	295.8	6.6	287.8	45.2	4138	101	8	0.2
1570	156.7	3.9	138.6	6.1	2882	107	18.1	0.6
255	496	40.4	634.4	68.8	8632	484	−138.4	−1.6

Errors in allometric AGB for buttressed trees occur due to the limitations in the allometric data and variations in trunk form, but also because DBH measurements for these trees have to be made above the buttresses and hence do not represent ‘true’ DBH. Above-buttress measurements are typically noted in manual surveys, and empirical correction factors may be applied *post facto* where appropriate. These errors are avoided in the TLS approach, and so TLS measurements of buttressed trees could be used to correct existing ASEs and to characterize the number (and size) of buttressed trees in sample plots. This would allow existing ASEs to better represent the allometry of buttressed and irregular trees and reduce uncertainty in the resulting AGB estimates at large scales.

### 3.5. Separating leaf and wood in terrestrial laser scanning point clouds

Another important uncertainty in TLS-derived estimates of AGB is that these methods typically require wood-only points (although QSM fitting has been done leaf-on with small leaves, e.g. [49]). However, many other properties we might derive from TLS require both leaf and wood returns. Methods to quantify biophysical forest properties such as gap fraction, plant or leaf area index (PAI, LAI) etc. usually focus on either wood or leaf components [69,70]. Separating these components in TLS data is an ongoing challenge [56,71,72]. Two broad approaches have emerged: (i) methods exploiting differences in the return intensity of the signal and (ii) methods based on geometrical descriptors of the point cloud, i.e. assuming some *a priori* knowledge of how leaves and branches are co-located in 3D space. A somewhat different third approach uses volumetric estimates of leaf area density [73–75]. We differentiate this approach due to the slightly different interpretation of the resulting volumetric rather than explicit 3D leaf/wood area.

Various authors have proposed using dual- or multi-wavelength lidar [71,76–78] to exploit different material reflectance at different wavelengths. In practice, the widely varying orientation of canopy objects and partial lidar hits may overwhelm these differences. While leaf/wood separation methods based on multispectral lidar intensities are potentially promising, there are still many practical difficulties to overcome, not least calibration to provide physically meaningful return intensity values [79].

The second approach to leaf/wood separation is based on analysing the geometric properties of the point cloud and then classifying point clouds into their constituent materials based on geometric descriptors [72,80,81]. These methods differ from the more instrument-specific or intensity-driven approaches, in that they rely on machine learning algorithms to assign points to leaf and wood/other classes based on location in relation to other points and canopy elements, or clustering according to the point cloud structure [82]. These various methods have shown promise and are potentially applicable to virtually any tree point cloud. The chief drawback is the (typical) requirement for manual input to filter individual point clouds, which is impractical for processing large numbers (hundreds or thousands) of trees.

**Figure 9.**
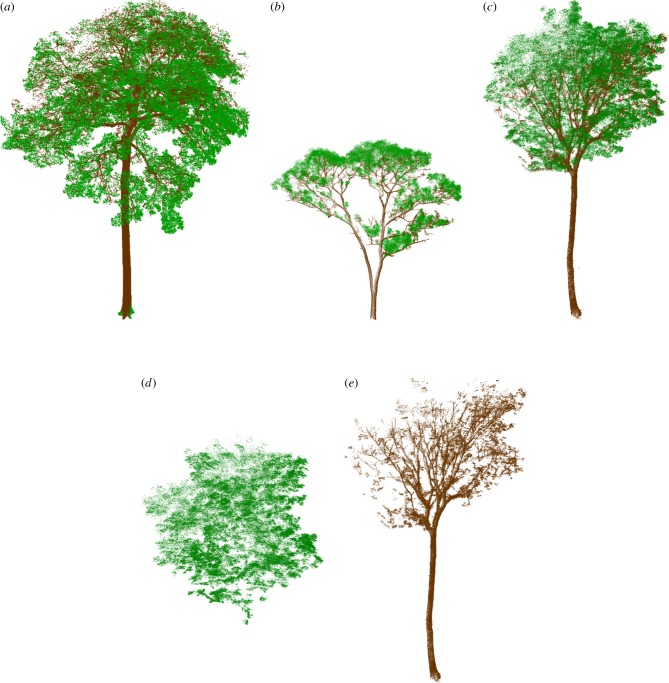
Examples of leaf/wood separation algorithm applied to trees of varying type, using the framework developed by Boni Vicari [82,83]. (*a*–*c*) A single ash tree from Wytham Woods [45,46]: (*a*) leaf and wood material; (*b*) leaf points only; (*c*) wood points only. (*d*,*e*) Individual trees with leaf (green) and wood (brown) material separated: (*d*) tropical hardwood (sp. unknown) from Nouragues, French Guiana [15]; (*e*) eucalypt (*Eucalyptus leucoxylon*), from Rushworth forest, Victoria, Australia [51].

Figure 9 shows results from applying a leaf/wood separation algorithm developed by Boni Vicari [82]^3^ from the method of [80] and [81]. The algorithm uses a shortest path approach to detect the trunk and larger branches, following which an unsupervised classification is applied to the remaining points. This is based on 3D geometric descriptors calculated using the nearest neighbours of each point and then applying Gaussian mixture models with an expectation/maximization algorithm. The results in figure 9 are from trees of varying leaf and branch properties collected in very different environments. While the separation examples certainly look plausible (and initial tests suggest they are), this illustrates one of the key limitations of many of the methods used to estimate tree and forest structural and biophysical parameters: the problem of validation.

### 3.6. Validation

True ‘validation’ of estimates of tree properties (such as volume, AGB and leaf/wood material no matter how they are derived) can be achieved only by destructive sampling. As outlined above, this is either too expensive and time-consuming or is simply not possible or desirable. The problem of validating wood and crown volume estimates from TLS has been noted [84,85]. True validation of TLS-derived AGB (or allometric for that matter) requires measurement of the volume of trees that have been scanned and reconstructed. This implies destructive harvesting and measuring of wood volume and/or mass (wet and dry). Lack of destructive harvest data is perhaps the largest uncertainty in ASEs [6,12–14]. It is also why validating volume reconstruction has often been limited to a combination of internal consistency checks and visual inspection [57,84,85]. Very few studies have compared destructive harvest volumes/AGB directly with QSM-derived estimates from TLS. The two most comprehensive comparisons, comprising 95 trees in total from temperate eucalypt [49] and tropical forest [25], found that TLS-derived estimates of AGB agreed with harvested AGB with *r*^2^ > 0.97.

The same difficulty of validation arises for leaf/wood separation algorithms: lack of harvest data makes validation near impossible, so how *can* we validate a particular algorithm? Deciduous woodland presents one very effective way, by allowing TLS scans made at the same locations under leaf-on and leaf-off conditions to be compared [44,45]. However, this approach is time-consuming in its own right, requiring precise comparison of scans across seasons, when other changes may also occur in the interim. It is also limited, by definition, to deciduous woodlands.

Another validation strategy, not just for volume and AGB, but also leaf/wood separation, is the use of highly detailed 3D tree structural models [86–88]. 3D radiative transfer (RT) models have been developed to produce very accurate simulated TLS point clouds from 3D structural models. Arbitrary reconstruction models or leaf/wood separation methods can then be applied to the simulated point clouds and the results can be validated accurately, given that the 3D tree structural details are specified *a priori* [53,56–58,85,89,90]. Boni Vicari [82] has developed a generic testing framework to allow leaf/wood separation algorithms in this way, available from Boni Vicari [83]. The 3D RT approach can also be used to explore arbitrary TLS instrument properties and data collection protocols [46]. This type of approach has been used to help extend the QSM approach to explore how leaves and needles might be added to QSMs in a realistic way [56].

The drawback of the 3D RT model approach to ‘validation’ of TLS reconstruction is that the issue then arises of how realistic the driving 3D structural models are. However, as more high-quality TLS data are collected and used to generate (validated) QSMs, the more feasible it becomes to use these as inputs for the 3D RT model simulation and QSM testing. A caveat is that care must be taken to avoid circularity, i.e. not testing a 3D reconstruction approach using a simulated point cloud derived from the same, or similar, 3D reconstruction method.

### 3.7. Terrestrial laser scanning and allometric scaling equations

Empirical ASEs are currently the only way to extrapolate plot-scale tree measurements to larger areas. These range from locally calibrated or species-specific ASEs to those used for pan-tropical AGB estimates from remote sensing [6,10,11]. Uncertainty in the resulting AGB estimates arises, in large part, because the ASEs are used to extrapolate small samples of destructively harvested trees to a range of forest types, and, crucially, trees with larger diameter that are poorly sampled in the harvest data. This can lead to large out-of-sample extrapolation errors [50]. A meta-analysis by Sileshi [13] of over 600 published ASEs showed that 60% were derived from samples of fewer than 30 trees, only 20% contained samples of more than 50 trees, and uncertainty was rarely considered [16,91,92]. Sampling bias was also apparent in the broad genera of trees harvested for ASEs, i.e. oversampling of dipterocarps (smaller crown/DBH) versus legumes (larger crown/DBH).

This lack of large trees in allometric samples leads to large uncertainties in AGB, due to the disproportionate biomass of large trees [6]. This problem is compounded because the distribution of large tree AGB is heteroscedastic, i.e. trees exhibit increasing variation in AGB with increasing diameter [93]. This implies that minimizing ASE uncertainty requires more destructive samples of larger trees than smaller ones; in practice, the opposite is the case. TLS can address this limitation by providing volume estimates across all sizes, without size bias. TLS can also provide accurate *H* for all trees, unlike census-based measurements, meaning that TLS-derived ASEs ought to be robust to variations in canopy DBH : *H*. Recent work shows that the size bias in pan-tropical ASEs, for example, can be overcome using TLS measurements [15].

An additional, important uncertainty in allometric estimates of AGB is wood density, *ρ*. *ρ* varies both within and between species and/or region [14,94–97] as well as radially and with height in individual trees. Intra- and interspecific differences in *ρ* arise in part because it is a strong determinant of mechanical support [98], but also due to differences in environmental and evolutionary strategies, or phylogeny [99–101]. Variations of *ρ* (and their treatment in ASEs) have been proposed as an explanation for the large observed differences between pan-tropical AGB estimates [28,29]. While TLS measurements cannot address *ρ* variation directly, TLS-derived QSMs can be used to quantify the sensitivity of AGB estimates to variations in *ρ*, by varying *ρ* as a function of height, branching order and branch radius within a QSM.

### 3.8. Uncertainty in allometric model form

Limitations in allometric data lead to uncertainties in ASEs which are poorly characterized [16,102,103]. ASE form is also a major determinant of the resulting uncertainty (and bias) of AGB estimates, but is also poorly understood and often overlooked [6,7,13,104]. ASE models are mostly fitted by log-transformed ordinary least-squares regression, which relies on the assumptions of homoscedasticity and normality in the underlying data [93]. Given that measured allometric data rarely if ever conform to these assumptions, this log-transformation is another potential source of uncertainty, particularly systematic bias. Recent work has shown how uncertainty in allometric estimates of AGB grows rapidly with tree size due to these ASE modelling assumptions [15,50]. Once again, this is an area where TLS can prove invaluable, by providing many more samples of tree-level AGB, with well-quantified uncertainty, particularly from larger trees. Crucially, the resulting TLS estimates of AGB are independent of allometric models.

### 3.9. Conclusion: challenges and opportunities

New TLS-derived measurements of 3D structure have the capability to transform estimates of AGB. TLS measurements can address key uncertainties in allometric estimates of AGB, particularly tree shape, size bias in allometric samples, and enable better quantification of errors due to wood density *ρ* and ASE model form. The independence of TLS-derived AGB estimates from allometry is a huge benefit in this regard. TLS data also provide accurate estimates of tree height *H*, which are needed for improved calibration and validation of remote sensing estimates of AGB, which rely almost exclusively on *H*-based allometries of one form or another.

If the accuracy of TLS-derived estimates of AGB is demonstrated across a wide range of tree species and forest types, they are likely to become invaluable for improved monitoring of C stocks and fluxes. This is particularly important for international forest monitoring and protection agreements [33,34]. TLS-derived estimates of AGB can potentially revolutionize our understanding of C stocks and fluxes in the tropics [15]. Acceptance of TLS measurements for wider monitoring strategies will require additional destructive harvesting of scanned trees across multiple biomes, as well as much wider availability of TLS tools and methods that are more readily accessible to forestry and field researchers. Perhaps even more important is the need for corresponding developments in training and education.

A note of caution is also required. TLS methods cannot replace empirical allometric methods, particularly remote sensing measurements. The requirements of time and manpower mean that, currently, TLS collection is only really feasible at the same sort of scale as field-based survey/census measurements. A single hectare plot takes 3–6 person days to scan at high detail (depending on terrain and instrument properties and accuracy requirements), i.e. around the same time as a typical field census survey. However, processing the TLS data into useful quantitative structural information for AGB estimates etc. requires the same or more time, for co-registration, tree extraction and QSM reconstruction [46]. An important part of this process is that traceability of uncertainty in the resulting QSM reconstruction can be included in the processing chain.

This whole process depends on access to expensive capital equipment (the lidar instruments themselves), deployment costs, high levels of expertise, significant computing power and potentially expensive software. As a result, these methods are currently out of reach for many researchers. The advent of smaller, lower-cost TLS instruments show a lot of promise [105], although their use for the accurate 3D forest measurement required for QSM methods has yet to be validated (with destructive sampling) given their typically much lower range and precision. Increased availability of TLS data of all kinds, as well as the proliferation of point clouds from unmanned aerial vehicles and shape-from-motion techniques is leading to the development of new, open source software tools. This will facilitate wider access to TLS modelling [106–108]. If measurement protocols can be standardized, this will also increase the uptake of TLS for AGB and other applications [46].

Finally, reliable TLS measurements of 3D tree structure can provide advances far beyond just AGB [52,109]. In terms of biomass, below-ground measurements of tree roots are much harder again to make than even above-ground measurements. Initial work has shown that it is feasible to make TLS-derived estimates of below-ground biomass in much the same way as for AGB, but at significant extra effort [110,111]. More generally, TLS is providing new 3D structural measurements for exploration of tree structure form and function at a fundamental level. Large quantities of TLS data of individual 3D tree structure will open new and exciting avenues of research in areas where the difficulty of measurement has until now prevented large-scale statistical approaches to detecting and understanding underlying patterns of scaling, form and function [52].

## Endnotes

1 In a direct sense. Below-ground biomass is even harder to measure than AGB (and hence less frequently done), requiring huge manual effort to expose and remove potentially very large root systems.

2 The TreeQSM code is available via Github (https://github.com/InverseTampere/TreeQSM), and from the author Dr Pasi Raumonen (pasi.raumonen@tut.fi).

3 Code available via: https://github.com/mattbv/tlseparation.

## Data accessibility

This article has no additional data.
